# Adaptation of a fluoroquinolone-sensitive *Shigella sonnei* to norfloxacin exposure

**DOI:** 10.1098/rsos.232025

**Published:** 2024-06-19

**Authors:** Bao Chi Wong, Soffi Kei Kei Law, Muhammad Zarul Hanifah Md Zoqratt, Qasim Ayub, Hock Siew Tan

**Affiliations:** ^1^ School of Science, Monash University Malaysia, 47500 Bandar Sunway, Selangor Darul Ehsan, Malaysia; ^2^ Monash University Malaysia Genomics Platform, 47500 Bandar Sunway, Selangor Darul Ehsan, Malaysia; ^3^ Tropical Medicine and Biology Multidisciplinary Platform, 47500 Bandar Sunway, Selangor Darul Ehsan, Malaysia

**Keywords:** *Shigella sonnei*, whole-genome sequencing, transcriptome analysis, sublethal conditions

## Abstract

*Shigella* causes shigellosis that requires antibiotic treatment in severe cases. Sublethal antibiotic concentrations can promote resistance, but their effect on antibiotic-sensitive bacteria before resistance development is unclear. This study investigated the effects of sublethal norfloxacin (NOR) challenges on a NOR-sensitive strain, *Shigella sonnei* UKMCC1015. Firstly, the whole genome of *S. sonnei* UKMCC1015 was assembled, and 45 antimicrobial resistance (AMR) genes were identified. Interestingly, transcriptomic analysis showed that low NOR levels do not change either the expression of the AMR genes or NOR targets such as *gyrA*. Instead, multiple ribosomal protein genes were downregulated, which could be attributed to decreased ribosomal protein promoter activity, modulated by elevated guanosine pentaphosphate and tetraphosphate (ppGpp) levels. This alarmone is involved in the bacterial stringent response during environmental stress, and it is mainly produced from the ppGpp synthetase (*relA*). Additionally, we observed that a *relA* overexpression (prolonged period of elevated ppGpp levels) may negatively affect the NOR tolerance of the bacteria. In conclusion, this study revealed that a NOR-sensitive strain responds differently to sublethal NOR than commonly reported in resistant strains.

## 1. Introduction

*Shigella* species are common causal agents of episodic global diarrhoea, affecting mainly children younger than 5 years [1]. *Shigella flexneri* is commonly isolated in low-income countries, while *Shigella sonnei* is isolated mostly in high-income countries. During recent decades, *S. sonnei* has replaced *S. flexneri* as the dominant cause of bacillary dysentery globally [2]. The current population of *S. sonnei* descended from a common ancestor that led to distinct lineages and spread to other parts of the world, with strains in Asia that are mostly descendants of one lineage from Europe [3]. The etiological shift of shigellosis to *S. sonnei* has also been reported in Malaysia [4,5]. Most studies on *Shigella* in Malaysia have focused on its antimicrobial susceptibility profiles, and there is a lack of data on its genetics and phylogeny.

The misuse and overuse of antibiotics have resulted in an increased concentration of antibiotics in the environment, exacerbating the antimicrobial resistance (AMR) crisis [6]. Among all antimicrobial-resistant bacteria, fluoroquinolone-resistant *Shigella* spp. is the priority three (medium) category for new antibiotics research and development [7]. Studies have shown that low antibiotic concentrations might contribute to AMR [8–10]. Antibiotic concentrations higher than the minimum inhibitory concentration (MIC) lead to antimicrobial effects on susceptible cells. However, concentrations lower than the MIC (also known as sub-MIC) can act as a signal for diverse responses that ultimately induce tolerance [11]. As antibiotic pollution leads to sub-MIC levels of antibiotics in the environment, this condition can enrich pre-existing resistant variants and cause selective pressure to develop *de novo* resistance [12]. Thus, the effect of sub-MIC on antibiotic tolerance in sensitive bacterial strains needs to be well understood to counteract the inevitable presence of resistant strains.

Norfloxacin (NOR) is one of the first fluoroquinolones synthesized. Its mechanism of action is similar to ciprofloxacin, both of which are used to treat shigellosis. All fluoroquinolones act by inhibiting DNA gyrase and topoisomerase IV. Fluoroquinolones bind at the enzyme–DNA interface, blocking ligation of the DNA and leading to lethal chromosomal breaks [13]. By inhibiting bacterial DNA synthesis, it will ultimately cause bacterial cell death. Exposure to sub-MIC fluoroquinolone can promote resistance via mutations in the DNA gyrase gene [14]. However, the transcriptional responses of a sensitive bacterium to low levels of fluoroquinolone before developing AMR are not well characterized.

The main objective of this study is to determine the response of a fluoroquinolone-sensitive *S. sonnei* to a sublethal level of a first-generation fluoroquinolone NOR. We present the first circularized genome and characterize the antibiotic susceptibility profile of *S. sonnei* from Malaysia. Then, the bacteria was exposed to sublethal dose of NOR, and the transcriptomic response was analysed. The genes encoding the NOR target (DNA gyrase) are not differentially expressed. Instead, multiple ribosomal protein (r-protein) operons are significantly downregulated. We observe a slight decrease in several r-protein promoters and postulate that the alarmone guanosine tetraphosphate and guanosine pentaphosphate alarmone, collectively referred to as (p)ppGpp (ppGpp from here on), may be involved in the r-protein inhibition upon short exposure to sublethal NOR. This is because the elevated levels of ppGpp lead to the downregulation of multiple r-protein operons [15]. Thus, to further investigate the role of ppGpp in long-term NOR exposure, we evolve UKMCC1015 *relA* mutants (with different intrinsic levels of ppGpp) in lethal doses of NOR using the adaptive laboratory evolution approach. Without *relA*, NOR resistance requires mutations in the DNA gyrase. Additionally, we observe that overexpression of *relA* confers moderate NOR resistance without mutating the DNA gyrase.

## 2. Material and methods

### (a) Strain growth and culture conditions

Bacterial strains and plasmids used in this study are listed in the electronic supplementary material, table S1. *Shigella sonnei* UKMCC1015 strain is a clinical isolate purchased from the University Kebangsaan Malaysia Culture Collection (UKMCC). *Escherichia coli* DH10β was used for general cloning purposes. All the strains used in this study were cultured in Mueller–Hinton broth (Oxoid, UK) and incubated at 37°C with shaking at 200 r.p.m. overnight. When necessary, 50 µg/ml of kanamycin, 100 µg/ml of ampicillin (AMP) and 15 µg/ml of chloramphenicol (CHL) were added to the culture media for selection. Total genomic DNA was extracted from the mid-log culture using the Wizard® Genomic DNA Purification Kit from Promega following the manufacturer’s protocol.

### (b) Genome sequencing, assembly and annotation

We used a hybrid approach to obtain the complete genome, employing long-read Oxford Nanopore Technology and short-read Illumina MiSeq sequencing. Briefly, for long-read sequencing, DNA libraries were prepared using the Ligation Sequencing Kit (SQK-LSK109) according to the manufacturer’s protocol and sequenced on a MinION FLOW-MIN106 flowcell and MinION MK1B sequencing device (Oxford Nanopore Technologies). Base-calling was conducted with Guppy v. 3.2.10 on MinKnow 3.6.17 using a fast base-calling configuration. For Illumina MiSeq sequencing, DNA libraries were prepared using the Nextera XT library preparation kit and sequenced using a 2 × 250 bp paired-end configuration. The Illumina reads were trimmed using Trimmomatic v. 0.39 [16] and used for error correction using Pilon v. 1.23 [17]. For the genome assembly, multiple long-read assemblers were used: Flye v. 2.6 [18], Raven v. 1.1.10 (https://github.com/lbcb-sci/raven) and Miniasm-Minipolish v. 0.3 (https://github.com/rrwick/Minipolish). Then, Trycycler (https://github.com/rrwick/Trycycler) was used to obtain the consensus long-read assemblies from different long-read assemblers. The long reads were also used for error correction using Medaka v. 1.0.3 (https://github.com/nanoporetech/medaka). The genome was annotated using the Prokaryotic Genome Annotation Pipeline (PGAP) developed by the National Center for Biotechnology Information (NCBI) [19]. Additionally, AMR genes were annotated using the Comprehensive Antibiotic Resistance Database (CARD) [20]. The genome and plasmids in UKMCC1015 were constructed using Basic Local Alignment Search Tool (BLAST) Ring Image Generator (BRIG) [21].

### (c) Antimicrobial susceptibility testing and bacterial growth under different concentrations of NOR

The antimicrobial susceptibility profile of the UKMCC1015 strain was determined by the agar disc-diffusion method. The results were interpreted according to the Clinical and Laboratory Standards Institute (CLSI) guidelines. The NOR MIC for UKMCC1015 strains was determined using the broth-microdilution method. *Escherichia coli* 25922 was used as a reference control. The growth curve of UKMCC1015 was determined using 0×, 0.2×, 0.5× and 1.0× NOR MIC. The overnight culture of UKMCC1015 was diluted to an initial optical density (OD 600 nm) of 0.05 in 50 ml of broth containing the different antibiotic concentrations with shaking at 200 r.p.m. The OD 600 nm was obtained every 15 min for 5.5 h.

### (d) Bacterial growth and RNA extraction

To study the short-term effect of a low dose of NOR on the transcriptome of UKMCC1015, overnight culture was diluted and allowed to grow in the presence of 0.2× MIC NOR (37°C with shaking at 200 r.p.m.) until the mid-log phase (OD 600 nm of 0.50) is reached. Cultures grown in the absence of NOR were used as controls. RNA was extracted from the bacterial cells using the TRIzol phenol–chloroform extraction method according to the manufacturer’s protocol. rRNA depletion was performed using the MICROBExpress Bacterial mRNA Enrichment Kit (Thermo Fisher Scientific, MA, USA) following the manufacturer’s protocol and cDNA library preparation using the NEBNext Ultra RNA Library Prep Kit for Illumina (New England Biolabs, MA, USA). DNA library quality control was done by measuring concentration using the Qubit 2.0 fluorometer (Invitrogen, CA, USA) and assessing the size distribution using TapeStation 2200 (Agilent Technologies, CA, USA). All libraries were pooled equimolar, and 15 pM was loaded into the MiSeq cartridge. Sequencing was performed on the MiSeq system using a 150 bp run configuration.

### (e) RNA bioinformatics analyses

The *S. sonnei* UKMCC genome (GenBank assembly accession: GCA_014217935.1) was used as the reference genome to map the RNA-seq data. Genome annotation was obtained from the NCBI PGAP annotation. *Shigella sonnei* Ss046 ncRNA sequences from the Bacteria Small Regulatory RNA Database (BSRD) [22] were used to BLAST against the *S. sonnei* genome to find additional ncRNA genes in the genome. BLAST hits were then appended to the annotation. To properly merge the two annotation sources, if annotations of the two sources overlapped, the PGAP annotation took precedence over the BLAST BSRD hits.

Illumina adapter sequences and low-quality bases were trimmed off using Trimmomatic v. 0.39. The trimmed, quality-filtered reads were mapped to the reference genome using Bowtie2 v. 2.3.5 and SAMtools v. 1.9 [23,24]. The alignments on annotated genes were counted using htseq-count v. 0.11.2 [25]. Features included in the htseq counting were the coding sequences (CDS), rRNA, ncRNA, tRNA, antisense RNA and riboswitch. Differential abundance analyses were conducted using DESeq2 [26] on the Galaxy platform, while the visualization through the volcano plot was done using the web server VolcaNoseR [27]. The Clusters of Orthologous Genes (COGs) were determined using eggNOG (evolutionary genealogy of genes: Non-supervised Orthologous Groups), a web-based server [28]. Heatmaps were made using Python package seaborn [29] and Matplotlib [30].

### (f) Recombinant DNA techniques and oligonucleotides

Plasmid DNA and PCR products were extracted and purified using the FavorPrepTM Plasmid Extraction Mini Kit and the GEL/PCR Purification Kit according to the manufacturer’s instructions. For PCR, Q5® High-Fidelity, Taq DNA polymerases (New England Biolabs) and 2× Taq PCR MasterMix (SolarBio) were used according to the manufacturer’s recommendations. Cloning was performed using restriction enzymes (New England Biolabs & Vivantis Technologies), Quick CIP and T4 DNA ligase (New England Biolabs) described by the manufacturer. All primers used in this study were synthesized by Integrated DNA Technologies (IDT) (electronic supplementary material, table S2).

### (g) Construction of ribosomal promoter–reporter plasmids

Plasmids were constructed using the restriction enzyme-based cloning approach. The promoter replacement approach was applied to clone different r-protein promoters into the pUltra-RFP [31]. R-protein promoter sequences of 151 nucleotides directly upstream of the transcriptional start site (TSS) were used in this analysis. The TSS was identified based on the TSS in *E. coli* [15]. The r-protein promoters rpsJp, rpsLp, rpsMp, rpsPp, rplNp and rpsFp were amplified from UKMCC1015 gDNA and ligated to pUltra-RFP by EcoRI and PstI restriction sites. Plasmids constructed were transformed into *E. coli* DH10β via the heat-shock/calcium chloride procedure [32]. The constructs were verified by colony PCR and Sanger sequencing (IDT). The plasmids were extracted and transformed into UKMCC1015.

### (h) Fluorescence assay

To analyse the effect of NOR on the promoter activity of various r-protein promoters, overnight cultures of the UKMCC1015 mutants were diluted to a starting OD 600 nm of 0.05, and NOR, at a final concentration of 0.2× MIC, was added and allowed to grow until it reached the mid-log phase. Cultures without NOR stress served as controls. Then, the cells were spun down at 13 000 r.p.m. for 5 min. The cell pellets were resuspended in 1× phosphate-buffered saline of equal volume and vortexed. Then, 200 µl of the cells were added to a transparent, flat 96-well plate with a clear bottom. Relative fluorescence unit (RFU) was measured using the Infinite M200 (Tecan) microplate reader. Absorbance was measured at OD 600 nm, and the fluorescence signals were detected using 535 nm/620 nm (excitation/emission) and collected as top readings. Absorbance and fluorescence readings were corrected for background absorbance and fluorescence from the medium. The RFU value was normalized against the bacterial cell density (RFU/OD 600 nm) using the following formula:


$$\frac{RFU_{raw}-\;RFU_{medium}}{OD_{raw}-OD_{medium}}.$$

### (i) Construction of UKMCC1015 *∆rel*A knockout and *relA* overexpression mutant

The knockout mutant (UKMCC1015 *ΔrelA*) was constructed using homologous recombination. The wild-type UKMCC1015 (WT) was transformed with pGETrec plasmid, which carries the homologous recombination system, and selected using 100 µg/ml of AMP. DNA fragments with 300 bp homology arm upstream and downstream of *relA* were amplified. The CHL-resistant cassette was amplified from the pBeLoBAC11 plasmid. These three PCR fragments were subjected to overlap extension PCR to generate the *ΔrelA* homologous template. The template was gel-purified using Monarch PCR & DNA Cleanup Kit (New England Biolabs) and transformed into UKMCC1015 electrocompetent cells. In total, 1 ml of SOC medium was immediately added to the electroporated and incubated for 1 h at 37°C with shaking before plating the culture on CHL agar to select for recombinants. UKMCC1015 *ΔrelA* was verified by colony PCR and Sanger sequencing. The pGETrec plasmid was removed from UKMCC1015 *ΔrelA* by growing the strain for 4 days with 0.2% L-arabinose and without AMP. To construct the overexpression mutant (UKMCC1015 *relA* ^+^), *relA* was amplified from the UKMCC1015 gDNA and ligated to the pBAD24 vector, an arabinose-inducible plasmid [33], resulting in the construction of pBAD24_*relA*. This plasmid was transformed into *E. coli* DH10β and recovered on AMP agar. The plasmid was transformed into the UKMCC1015 after the construct was verified by colony PCR and Sanger sequencing (IDT).

### (j) Long-term and short-term NOR stress

Long-term NOR stress was when the bacteria was exposed to NOR for 14 days. This was used to evolve for NOR resistance in the UKMCC1015, UKMCC1015 *relA^+^* and UKMCC1015 *ΔrelA* mutants. Bacteria were grown in Mueller Hinton broth (MHB) at 37˚C with 200 r.p.m. shaking. Bacteria were subcultured using fresh MHB in the presence of 1× MIC NOR daily for 14 days. The culture was diluted by 40 000-fold, and 100 µl was plated on MH agar containing different concentrations of NOR. Several colonies were selected from the NOR agar plates, and the new MIC of these colonies were determined using the broth-microdilution method.

Short-term NOR stress was when the bacteria was exposed to NOR for 2 h. Overnight culture of UKMCC1015-evolved strains was diluted to an initial OD 600 nm of 0.05 and grown to mid-logarithmic phase (OD 600 nm of 0.5) at 37°C with shaking at 200 r.p.m. For UKMCC1015 *relA* ^+^, 0.2% of l-arabinose was added to the culture medium. At OD 600 nm of 0.5, the bacteria grew for another 2 h in the presence of 1× MIC NOR. Then, RNA was extracted as described earlier.

### (k) Determination of *relA* expression through semi-quantitative RT-PCR

Extracted RNA was treated with DNaseI and purified using a Monarch RNA Cleanup Kit (New England Biolabs). The cDNA was synthesized from approximately 1000 ng of RNA using ReverTra Ace qPCR RT Master Mix with gDNA remover (TOYOBO, Japan) following the manufacturer’s protocol. Approximately 100 ng of cDNA for each sample was used to determine *relA* expression (using 16S rRNA as a housekeeping gene). The *relA* (4 µl) and 16S (2 µl) PCR products were run on 2% tris-borate-EDTA (TBE) agarose gel, post-stained with ViSafe Red Gel Stain (Vivantis Technologies) and visualized using GelDoc (BioRad). The relative intensity of the bands was quantified using ImageJ and the expression of *relA* was normalized to the 16S rRNA expression levels.

## 3. Results

### (a) Whole-genome sequencing of *S. sonnei* UKMCC1015 revealed the presence of AMR genes

**Figure 1.**
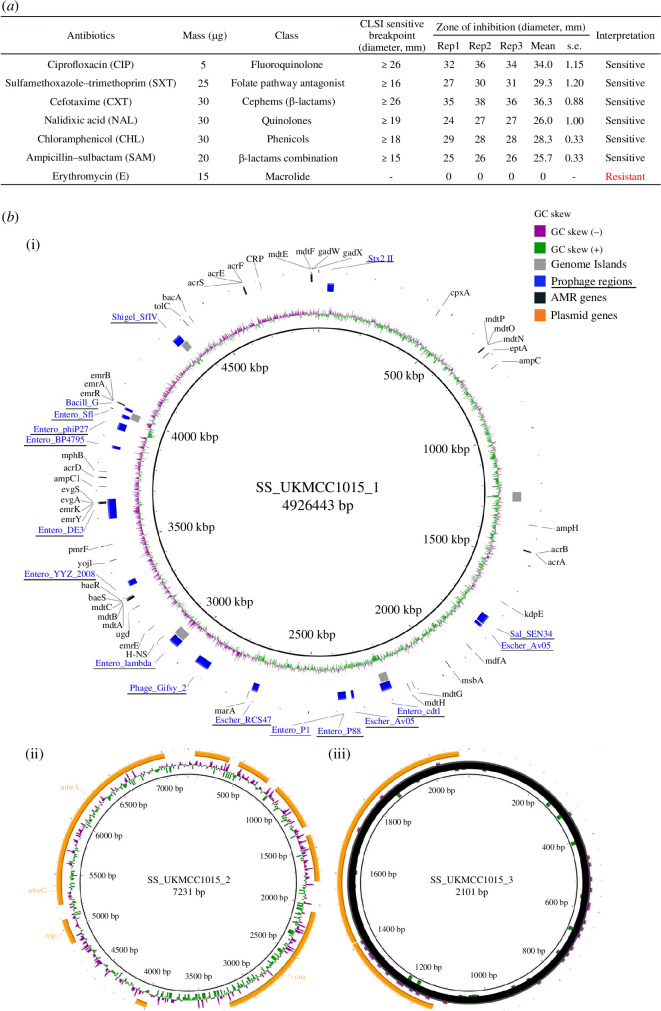
Phenotypic and genotypic characteristics of *Shigella sonnei* UKMCC1015. (*a*) Antibiotic susceptibility profile of *S. sonnei* UKMCC1015. (*b*) The closed chromosome of UKMCC1015, with UKMCC1015_1 (chromosome) and two plasmids (i), UKMCC1015_2 (ii) and UKMCC1015_3 (iii). UKMCC1015_1 chromosome, from inner ring outwards: GC skew, genome islands (grey, obtained from IslandViewer predicted with at least two prediction methods), Prophage regions (blue, underlined, obtained from Phaster), AMR genes (black, obtained from Abricate using databases CARD). Plasmids of UKMCC1015, from the inner ring outwards: GC content, GC skew, genes in orange.

We characterized the antimicrobial susceptibility profile and the complete genome of UKMCC1015 from Malaysia and investigated the transcriptomic changes of this bacterium to a sublethal NOR concentration. UKMCC1015 clinical strain was resistant to erythromycin but susceptible to other antibiotics such as ciprofloxacin, sulfamethoxazole–trimethoprim, AMP–sulbactam, cefotaxime, nalidixic acid, as determined from disc-diffusion assay (figure 1*a*). This strain was also sensitive to NOR (MIC of NOR = 0.03 µg/ml using the broth dilution method). Next, we investigated the presence of any AMR genes within the genome through whole-genome sequencing. A closed chromosome and two plasmids were obtained using hybrid assembly for UKMCC1015 (figure 1*b* and assembly information in electronic supplementary material, table S3). The chromosome has a size of 4 926 443 bp, while the two plasmids, UKMCC1015_2 and UKMCC1015_3, have sizes of 7231 and 2101 bp, respectively, with an average GC content of 51.03% for the genome. Interestingly, the large virulence plasmid (220 kb) is not detected from this sequencing. This may be owing to the loss of plasmid through laboratory culture, as reported previously [34], or this strain may not have this plasmid. Besides, 45 AMR genes associated with resistance to fluoroquinolone, macrolides and tetracycline, among many other antibiotics, were identified from the UKMCC1015 genome (figure 1*b* and electronic supplementary material, table S4). Most of the AMR genes (82%: 37/45 genes) encode for efflux pumps, while the others are responsible for antibiotic inactivation (four genes) and drug target alteration (four genes). Nineteen genes encoding for efflux pumps can potentially confer fluoroquinolone resistance. Besides, no mutations were observed in genes commonly associated with fluoroquinolone resistance: DNA gyrase A and B (*gyrA* and *gyrB*) and DNA topoisomerase IV (*parC* and *parE*).

### (b) Downregulation of translation in response to sublethal concentration of NOR

**Figure 2.**
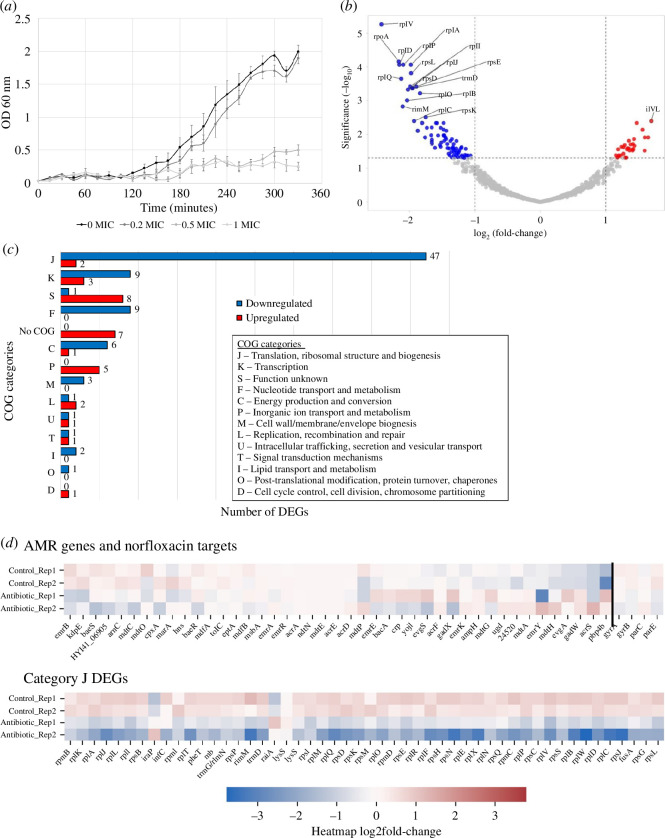
Effects of sub-MIC of NOR towards UKMCC1015. (*a*) Growth curve of UKMCC1015 WT under different sub-MIC of NOR (0×, 0.2×, 0.5× and 1.0× MIC). (*b*) Volcano plot of transcriptome analysis. Downregulated r-protein genes are represented by their respective abbreviations. (*c*) COG categorization of the 111 genes annotated by the non-supervised Orthologous Group (eggNOG). (*d*) Expressions of all AMR genes and NOR targets (*gyrA, gyrB, parC* and *parE*) that are not differentially expressed, and the expressions of the differentially expressed genes (DEGs) in category J (translation, ribosomal structure and biogenesis).

Based on the growth curve of UKMCC1015, 0.2× MIC NOR had the least effect on bacterial growth (figure 2*a*); hence, this sublethal NOR concentration was used in the subsequent RNA-sequencing (RNA-seq) analysis. The total RNA of UKMCC1015 grown on normal broth (control) and under 0.2× MIC NOR (experimental) was extracted, sequenced and analysed (electronic supplementary material, table S5). The control samples were distinct from the experimental samples from the principal component analysis (PCA) plot (electronic supplementary material, figure S1). A total of 112 differentially expressed genes (DEGs) were identified in response to the sublethal NOR challenge (adjusted *p*‐value ≤0.05, log_2_(fold-change) ≥ |1|). Thirty-one genes show greater than twofold upregulation, while 81 genes show greater than twofold downregulation (figure 2*b* and electronic supplementary material, table S6). Among these 112 genes, a non-coding RNA, *gcvB,* was differentially expressed. The remaining 111 genes were categorized using the COG database to elucidate further their role in UKMCC1015 response towards 0.2× MIC NOR (figure 2*c*). The top five categories were J (translation, ribosomal structure and biogenesis) with 49 genes, K (transcription) with 12 genes, S (function unknown) with nine genes, F (nucleotide transport and metabolism) with nine genes and C (energy production and conversion) with nine genes. None of the 45 AMR genes and the NOR targets found in UKMCC1015 were differentially expressed during 0.2× MIC NOR exposure (figure 2*d*).

**Figure 3.**
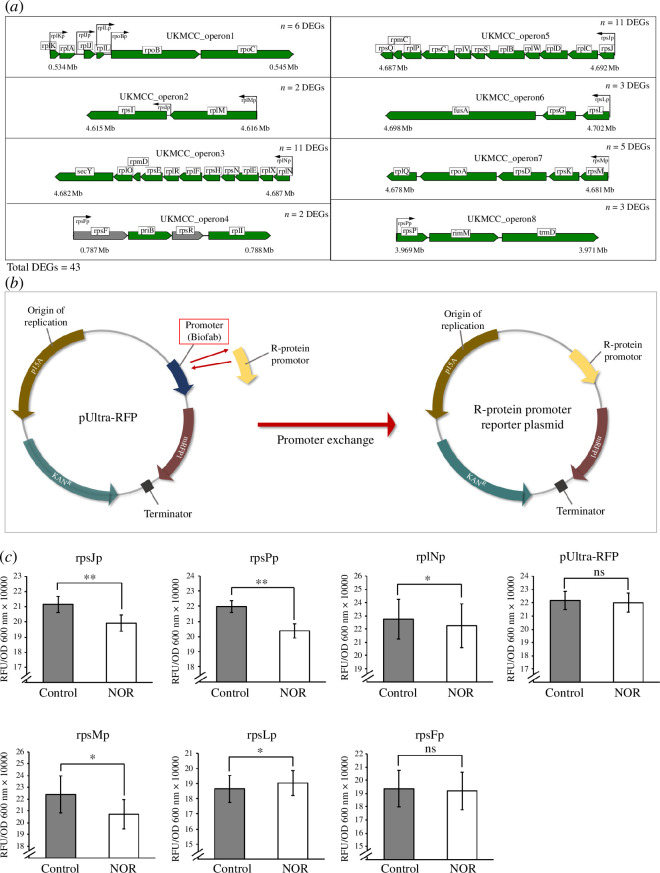
Schematic illustration of r-protein operons, promoter exchange approach and 0.2× MIC norfloxacin (NOR) effects on the promoter activity. (*a*) R-protein operons of UKMCC1015. Green indicates DEGs and grey indicates non-DEGs. (*b*) Construction of r-protein reporter plasmid via promoter exchange approach. (*c*) Normalized RFU/OD 600 nm changes for rpsJp, rpsMp, rpsPp and rpsLp, rplNp, rpsFp r-protein promoters and pUltra-RFP promoter in response to 0.2× MIC NOR stress. Three biological replicates were used for each promoter. Statistical significance (*p*‐value < 0.05) was determined using paired *t*‐test (one-tailed, *n* = 3); *p‐value < 0.05, **p‐value < 0.01, ns: non-significant.

Within category J, 47/49 DEGs were downregulated. The majority of the downregulated genes (37/47) encode r-proteins, which are involved in the assembly of the ribosomal subunits [35]. Given that r-proteins are commonly expressed in operons, in which multiple genes are co-transcribed [36], we postulated that the downregulation of the genes in category J could be owing to promoter regulation at the operon level. Indeed, 38/47 downregulated genes are expressed in eight operons (figure 3*a*), supporting our idea that any regulation of these genes could be done through the promoters.

To further validate our hypothesis, promoter–reporter fusions were constructed for several r-proteins (figure 3*b* and table 1), and the fluorescence readout from this reporter system was used to indirectly infer the activity of the r-protein promoters upon 0.2× MIC NOR exposure (figure 3*c*). Multiple promoters regulate the expression of operons 1 and 2. Therefore, these two operons are excluded from our analysis. Promoters from operons 3–8 were selected because these operons are under the control of only a single promoter (figure 3*a*), allowing direct measurements of the promoter activity on the operon transcription. The *rpsF* was not differentially expressed; hence, its promoter rpsFp was used as a control. The rpsJp, rpsPp, rpsMp, and rplNp promoters exhibited a slight decrease in the normalized RFU/OD 600 nm upon exposure to 0.2× MIC NOR with a percentage reduction of 5.81% (*p*‐value = 0.0018), 7.28% (*p*‐value = 0.004), 7.50% (*p*‐value = 0.036) and 2.17% (*p*‐value = 0.044), respectively, consistent with the RNA-seq findings, indicating that sublethal levels of NOR may reduce the activities of the r-protein promoters. The activity of the native promoter of the pUltra-RFP reporter plasmid that served as the negative control was also not significantly affected (electronic supplementary material, figure S2 and table 1).

**Table 1.** The log_2_(fold-change) and adjusted *p*-values of the r-proteins selected for validation.

R-protein gene	log_2_(fold-change)	adjusted *p*‐value
* rpsJ*	−1.7613	0.012333
* rpsP*	−1.23933	0.029703
*rplN*	−1.21306	0.025504
*rpsM*	−1.27704	0.034454
* rpsL*	−1.97617	0.000154
* rpsF*	−0.54654	0.455149052

### (c) ppGpp is not required for the evolution of NOR resistance

**Figure 4.**
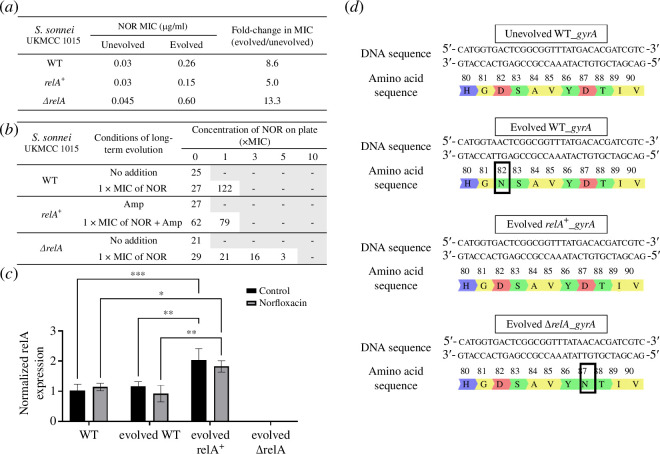
Response of UKMCC1015 and isogenic mutants to long-term exposure to NOR. (*a*) MIC values towards NOR for unevolved and evolved strains. (*b*) Actual number of colonies on agar containing different NOR concentrations after evolution. (*c*) Semi-quantitative PCR results of *relA* and 16S rRNA before and after short-term exposure to NOR. Statistical significance (*p*‐value < 0.05) was determined using two-way ANOVA; *p‐value < 0.05, **p‐value < 0.01, ***p‐value < 0.001, ****p‐value < 0.0001, and ns: non-significant. (*d*) Mutations at amino acid position 80–90 in gyrA for unevolved WT, evolved WT, evolved *relA^+^* and evolved Δ*relA*.

According to the RNA-seq data, r-protein genes were downregulated in response to NOR exposure. Besides, the past study reported that ppGpp could inhibit r-protein promoters [15]. Therefore, we hypothesized that sublethal NOR triggers the production of ppGpp and, in part, inhibits the expression of the r-proteins. To examine the contribution of ppGpp to the evolution of NOR resistance in UKMCC1015, we artificially overexpressed *relA* using the pBAD24 plasmid under the control of arabinose-inducible promoter. Besides, we constructed a UKMCC1015 *ΔrelA* mutant via homologous recombination to minimize the production of ppGpp. Overexpression of *relA* did not affect the intrinsic NOR resistance of UKMCC1015. However, the UKMCC1015 *ΔrelA* strain had a NOR MIC 1.5× fold higher than the WT, indicating that UKMCC1015 is more resistant to NOR when the ppGpp production is minimal (figure 4*a*).

We evolved the UKMCC1015 *relA^+^*, UKMCC1015 *ΔrelA* and UKMCC1015 WT (as a control) in 1X the respective NOR MIC for 14 days. The evolved cultures were plated on different concentrations of NOR. From the plating results (figure 4*b*), it can be seen that the *ΔrelA* mutant grew at a concentration of 5× MIC, unlike the WT and *relA^+^* mutant, which can only grow up to 1× MIC. The evolved WT, evolved UKMCC1015 *relA^+^* and evolved UKMCC1015 Δ*relA* had NOR MIC of 8.6-fold, 5-fold and 13.3-fold higher than their respective unevolved parental strain (figure 4*a*). This contradicts our initial hypotheses where the increase of ppGpp production is associated with the development of NOR resistance. We also observed that the evolved UKMCC1015 WT and evolved UKMCC1015 *ΔrelA* had single-nucleotide polymorphism (SNP) mutations in the genes encoding for DNA gyrase A (figure 4*d*). We identified D82N and D87N in *gyrA* in our evolved UKMCC1015 WT and evolved UKMCC1015 *ΔrelA* mutant, respectively. No SNP was identified in the *gyrA* of the evolved UKMCC1015 *relA^+^*.

Thus, we hypothesize that while ppGpp is needed during short-term exposure to NOR (around 2 h), the prolonged expression of ppGpp under long-term NOR stress is counterproductive to the evolution of NOR resistance. RelA produces ppGpp. Therefore, the expression of *relA* in the evolved strains was quantified using semi-quantitative RT-PCR to understand the role of ppGpp during short- and long-term NOR stress (figure 4*c* and electronic supplementary material, figure S3). The *relA* expression in evolved UKMCC1015 WT remains unchanged compared to unevolved WT. Overexpression of *relA* showed an approximately twofold increase in *relA* expression relative to WT, whereas UKMCC1015 *ΔrelA* did not express *relA*, as measured by semi-quantitative RT-qPCR. For short-term exposure to 1× MIC NOR, no significant changes were observed in the *relA* expression levels for all strains relative to the no-antibiotic control. This suggests that low intrinsic ppGpp levels exhibited higher NOR tolerance. However, a constant elevation of ppGpp production for a prolonged duration may reduce the bacterial tolerance to NOR.

## 4. Discussion

This study investigated the effects of sublethal exposure to a fluoroquinolone antibiotic, NOR, on *S. sonnei*, a pathogen responsible for shigellosis. UKMCC1015 was sensitive to most antibiotics tested, except for erythromycin, a macrolide antibiotic. As fluoroquinolones are the recommended treatment for shigellosis, NOR was chosen for this investigation. The results showed UKMCC1015 maintained the expressions of AMR genes and the NOR targets (DNA gyrase and topoisomerase IV) and downregulated the expression of the r-proteins leading to reductions in translation, transcription, energy production, and nucleotide production to cope with the sublethal NOR challenge.

Several past studies on fluoroquinolone-resistant *Shigella* indicate that the overexpression of genes encoding efflux pumps contributes to their resistance to fluoroquinolones [37–39]. Other responses towards fluoroquinolones include the downregulation of membrane porins to reduce membrane permeability and prevent antibiotic accumulation within the cell [40]. This suggests that the initial transcriptional response of fluoroquinolone-sensitive bacteria (UKMCC1015) is different from resistant ones. Instead of overexpressing the efflux pumps, the sensitive strain perhaps uses other pathways to cope with 0.2× MIC NOR. Additionally, there are no changes in the expressions of the NOR targets (DNA gyrase and topoisomerase IV). This may be owing to the main fluoroquinolone-resistance mechanism being linked to chromosomal mutations of *gyrA* and *parC* [40], which is not seen in UKMCC1015.

The transcriptomic analysis showed that exposure to sublethal NOR resulted in the underexpression of multiple r-protein operons even though r-proteins are not the NOR target. This observation was further supported using the promoter fluorescence assay. However, we observed only a slight reduction in the r-protein promoter activity compared to a larger reduction from the RNA-seq analysis, suggesting that a decrease in the r-protein promoter activities could partly drive the repression of multiple r-protein gene transcripts. Besides, the rpsLp promoter activity was significantly upregulated by 2.08%. This discrepancy could be owing to the delay between transcription and maturation of the fluorescent protein [41]. Overall, the large-scale downregulation of r-protein operons suggests that a common regulator may modulate the response of UKMCC1015 to sublethal NOR exposure.

One such regulator is the alarmone guanosine tetraphosphate and guanosine pentaphosphate alarmone, collectively referred to as (p)ppGpp (ppGpp). High ppGpp levels lead to the downregulation of multiple r-protein operons [15]. ppGpp is involved in the stringent response triggered during amino acid or fatty acid starvation or other environmental stress such as osmotic shock, heat shock, and oxygen variation [42]. Some roles of ppGpp include growth rate control by inhibiting the production of ribosomal RNA and the regulation of general metabolism through the expression of genes in amino acid biosynthesis [43]. Besides, ppGpp may mediate the downregulation of nucleotide synthesis and, subsequently, a decrease in DNA replication. As a result, the bacteria decrease the effect of NOR on the DNA gyrase and topoisomerase IV, enzymes involved in DNA replication. This is also supported by how *gyrA* and *parC,* the targets of NOR, are not upregulated during sublethal NOR treatment. Instead of increasing the number of targets and DNA replication to overwhelm the concentration of antibiotics, as seen in another study [44], this fluoroquinolone-sensitive UKMCC1015 decreases its activities and becomes semi-dormant.

Past studies showed that high levels of ppGpp help in antibiotic tolerance [45]. Hence, we investigated the role of ppGpp in modulating the adaptation of UKMCC1015 in response to long-term NOR exposure. ppGpp is synthesized/hydrolysed by the RelA/SpoT homologues superfamily proteins. The gene *relA* encodes for ppGpp synthetase with a single function, while *spoT* encodes for a bifunctional enzyme that mainly hydrolyses ppGpp with weak synthetase activity synthesis [46]. Given that the levels of ppGpp can be manipulated by overexpressing or deleting *relA*, the overexpression UKMCC1015 *relA^+^* and knockout UKMCC1015 *ΔrelA* mutants were constructed to investigate the role of ppGpp in the long-term evolution of NOR resistance. Interestingly, we observed that UKMCC1015 *ΔrelA* has a slightly higher NOR MIC than the WT. The NOR MIC for UKMCC1015 *relA^+^*, on the other hand, is similar to the WT. These findings indicate that without *relA*, UKMCC1015 developed a slightly higher intrinsic resistance to NOR, similar to a study that found an increase in AMP tolerance in an *E. coli ΔrelA* mutant in a minimal medium containing glucose after a prolonged stationary phase [47]. Others, however, have shown that knocking out *relA* leads to the loss of tolerance to AMP [48,49], mecillinam, [50] and microcin J25 [51]. This illustrates that ppGpp and stringent response may alter several aspects of antibiotic tolerance that lead to different tolerance levels.

Multiple studies reported the association between ppGpp and antibiotic tolerance/resistance. However, most were conducted quickly (within 48 h) [49,51–53]. We further showed that the evolution of UKMCC1015 WT and its isogenic mutants (UKMCC1015 *relA^+^* and UKMCC1015 Δ*relA*) upon exposure to 1× MIC NOR (14 days) led to NOR resistance mainly via mutation in *gyrA* irrespective of ppGpp level. The intrinsic *relA* expression levels remain roughly constant between the unevolved and evolved strains, suggesting that ppGpp does not promote the long-term evolution of UKMCC1015 in this experimental condition. Instead, the specific mutation in the *gyrA* likely affects the degree of NOR resistance. The absence of *relA* led to a higher NOR MIC in the evolved UKMCC1015 Δ*relA*, approximately 2.3-fold higher than the evolved WT. This UKMCC1015 *ΔrelA* developed a single mutation at position 87 (D87N), and this SNP has been previously associated with fluoroquinolone resistance [54–56]. In evolved WT, we identified a mutation D82N. This previously unknown mutation may contribute to the 8.6-fold increase in NOR MIC because the SNP is located near one of the quinolone resistance-determining regions (Ser83). High ppGpp levels activate stringent responses as a first-line defence to cope with stressful conditions such as antibiotic exposure. Our study, however, shows that without *relA* (minimal ppGpp production), *gyrA* mutation could be the main pathway driving the evolution of NOR resistance in UKMCC1015. Interestingly, no mutation occurs in the UKMCC1015 *relA^+^* overexpression strain, yet we observed a moderate increase in NOR resistance (5-fold). Given that stringent response slows down DNA replication, artificially activating and maintaining stringent response (through *relA* overexpression) may hinder the development of resistance mutations because stringent response slows down DNA replication [57]. Therefore, artificially upregulating the expression of *relA* in our study could activate other regulatory mechanisms, resulting in a moderate increase in the NOR MIC.

Our findings suggest that the constant elevation of *relA* expression (high ppGpp level) is counterproductive to developing NOR resistance. It is hypothesized that the increase in ppGpp owing to NOR exposure first slows down bacterial growth and translation as a transient response. ppGpp promotes DNA double-strand break repair by stabilizing backtracked RNA polymerase at the site of DNA breakage [58]. This allows the bacteria to develop NOR tolerance within the first few hours of exposure. However, increased ppGpp levels suppress bacterial growth, which is detrimental to bacterial survival. We postulate that high levels of ppGpp are beneficial during the initial stage of NOR exposure to allow for NOR tolerance but are detrimental in the long term. Thus, after 14 days of evolution, the UKMCC1015 *relA^+^* strain exhibits a lower proportion of survival at greater NOR concentration and a lower MIC than UKMCC1015 WT. In contrast, with reduced ppGpp levels, the *ΔrelA* mutant survives better under long-term exposure to NOR, with a higher MIC than the WT.

## 5. Conclusion

Overall, our genomic and transcriptomic studies provide new insights into how a fluoroquinolone-sensitive *S. sonnei* UKMCC1015 responds to a sublethal NOR challenge. The commonly reported NOR targets (DNA gyrase and topoisomerase IV) and AMR genes were not differentially expressed. However, many r-protein genes were downregulated, which may represent a consequence of the activation of ppGpp-associated stringent response. This study provides new insights into the multitude of responses *S. sonnei* can have to a low concentration of antibiotics, including the involvement of the stringent response and the alarmone ppGpp. Our study highlights that the antimicrobial response of a sensitive strain to a sublethal antibiotic challenge differs from that of a resistant strain. Additionally, a prolonged period of elevated ppGpp levels may negatively affect the NOR tolerance of the bacteria. However, strains with low levels of intrinsic ppGpp exhibited higher NOR tolerance. Future studies should focus on the exact pathways involving the stringent response that can aid in discovering potential targets for novel antibiotics or anti-resistance agents.

## Data Availability

The complete genome of *Shigella sonnei* UKMCC1015 and its plasmids have been deposited at GenBank under accession numbers CP060117, CP060118 and CP060119. RNA-sequencing data are accessible through BioSample accession number SAMN16871737. Supplementary material is available online [59].
